# A 36,000-Year-Old Volcanic Eruption Depicted in the Chauvet-Pont d’Arc Cave (Ardèche, France)?

**DOI:** 10.1371/journal.pone.0146621

**Published:** 2016-01-08

**Authors:** Sébastien Nomade, Dominique Genty, Romain Sasco, Vincent Scao, Valérie Féruglio, Dominique Baffier, Hervé Guillou, Camille Bourdier, Hélène Valladas, Edouard Reigner, Evelyne Debard, Jean–François Pastre, Jean-Michel Geneste

**Affiliations:** 1 Laboratoire des Sciences du Climat et de L’Environnement, UMR8212, LSCE/IPSL, CEA-CNRS-UVSQ, Université Paris-Saclay, Gif-Sur-Yvette, France; 2 Laboratoire PACEA, UMR 5199, CNRS, Université de Bordeaux, Pessac, France; 3 Laboratoire ArScAn, UMR 7041, Université Paris-Ouest Nanterre, Nanterre, France; 4 Laboratoire TRACES, UMR 5608 Université Toulouse-Jean Jaurès, Toulouse, France; 5 Laboratoire de Géologie de Lyon, UMR 5276 CNRS, Université Lyon1, Villeurbanne, France; 6 Laboratoire de Géographie Physique. UMR 8591 CNRS Universités de Paris I et XII, Meudon, France; 7 Centre National de la Préhistoire, Ministère de la Culture et de la Communication, Périgueux, France; University of Oxford, UNITED KINGDOM

## Abstract

Among the paintings and engravings found in the Chauvet-Pont d’Arc cave (Ardèche, France), several peculiar spray-shape signs have been previously described in the Megaloceros Gallery. Here we document the occurrence of strombolian volcanic activity located 35 km northwest of the cave, and visible from the hills above the cave entrance. The volcanic eruptions were dated, using ^40^Ar/^39^Ar, between 29 ± 10 ka and 35 ± 8 ka (2σ), which overlaps with the ^14^C AMS and thermoluminescence ages of the first Aurignacian occupations of the cave in the Megaloceros Gallery. Our work provides the first evidence of an intense volcanic activity between 40 and 30 ka in the Bas-Vivarais region, and it is very likely that Humans living in the Ardèche river area witnessed one or several eruptions. We propose that the spray-shape signs found in the Chauvet-Pont d’Arc cave could be the oldest known depiction of a volcanic eruption, predating by more than 34 ka the description by Pliny the Younger of the Vesuvius eruption (AD 79) and by 28 ka the Çatalhöyük mural discovered in central Turkey.

## Introduction

Volcanic eruptions are among the most impressive geological events on the surface of the earth. It is interesting to notice, however, that the oldest testimony of such an event in human history dates back only to about 9 ka [1]. Indeed it is only in 2013 that an Holocene eruption from the Hasan Däg (Central Turkey) dated at 8.97 ± 0.64 ka [1] strengthened the hypothesis made in the early 1960s by the archaeologist James Mallaart [2] that the Çatalhöyük mural coeval with the archeological level VII depicted a volcanic eruption. It has so far been considered the oldest known painting of a volcanic eruption. The second oldest one is found in Armenia but is more than 2 ka younger [3]. At this site, located in the Syunik upland, a group of six petroglyphs dated at 7 ka ago (5^th^ millennium BC) portrays the eruption of the Porak volcano [3]. These two sites predate by, at least, five millenniums the observations and testimony made by the Roman administrator and poet Pliny the younger of the AD 79 Vesuvius eruption (i.e. Letters 6.16 and 6.20).

About 340 Paleolithic caves with parietal art have been discovered in Europe, the large majority of them in South France and Northern Spain with the oldest dating back between 40 to 36 ka [4,5]. This period coincides with the arrival in Western Europe of anatomically modern humans (*Homo sapiens* [6]) and associated to the Aurignacian culture. The Upper Paleolithic European iconography combines figurative depictions of mainly wild animals with a predominance of herbivores (*e*.*g*. bison, horse, reindeer…) and less frequent human representations as well as diverse abstract patterns. Drawings of humans are usually schematic compared to quasi-naturalistic drawings of animals. So far, and despite the large number of caves studied since the early 19^th^ century, no painting, petroglyphs or engravings depicting natural scenery or geological phenomena from the Upper Paleolithic period have been found in Europe.

**Fig 1:**
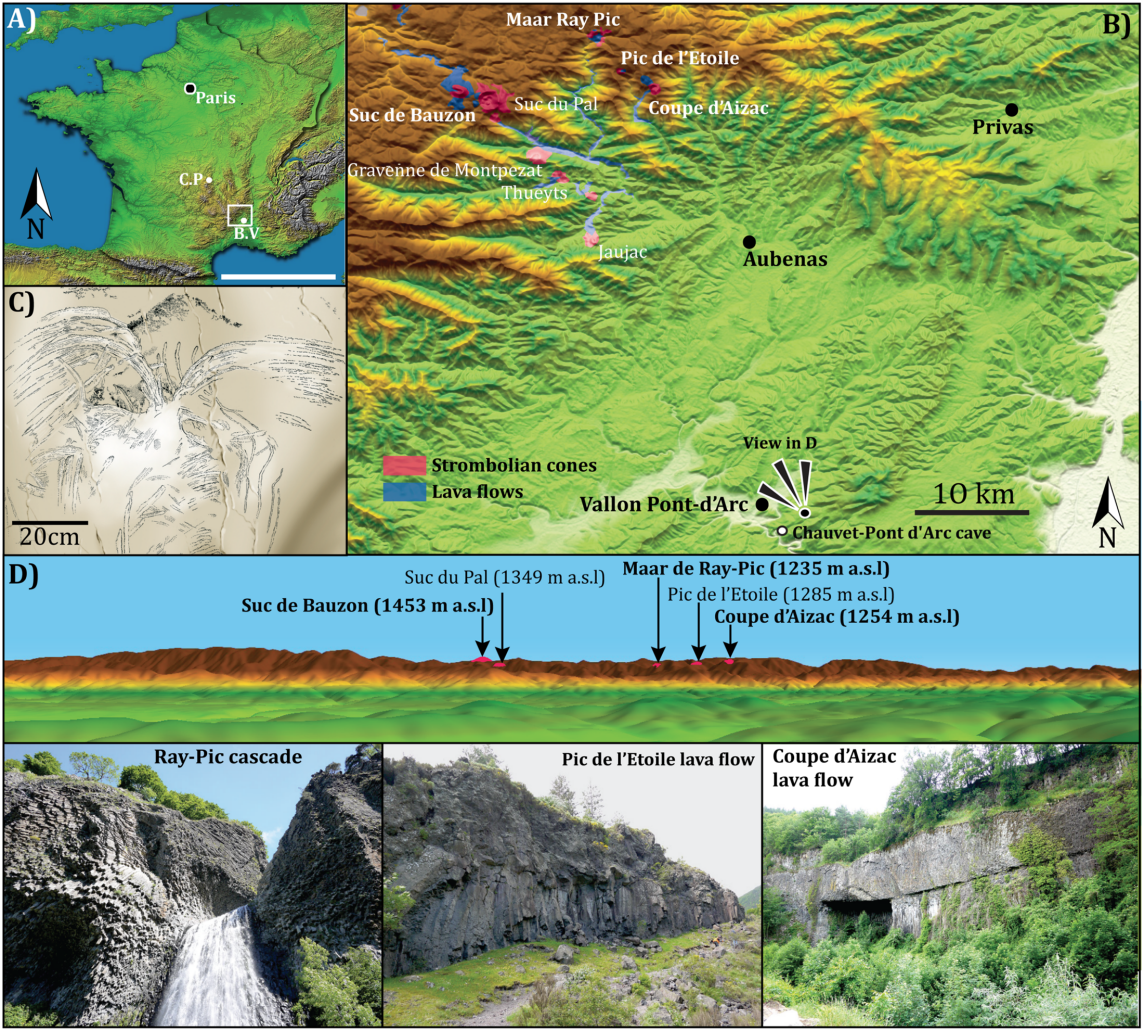
The Chauvet-Pont d’Arc cave and the Bas-Vivarais volcanic field. (A) Digital elevation model of France (Courtesy NASA/JPL-Caltech) showing the MIS 2–3 active volcanoes and the Chauvet-Pont d’Arc cave; C.P: Chaîne de Puys; B.V: Bas-Vivarais. (B) Digital elevation model of the Bas-Vivarais and Ardèche (Courtesy NASA/JPL-Caltech). The volcanic centers investigated are highlighted in bold italic fonts. (C) Detail of the spray-shape sign engraving from the Megaloceros panel. (D) View from the plateau above the Chauvet-Pont d’Arc cave showing several strombolian cones located 35 km Northwest (Courtesy NASA/JPL-Caltech).

Discovered on December 18, 1994, the Chauvet-Pont d’Arc cave (Ardèche, France; Fig 1) provides some of the earliest manifestations of prehistoric art and, as such, it was granted World heritage status by the United Nations (UNESCO) in 2014. Although the age of the paintings found in this cave has long remained controversial, radiocarbon dates have now robustly constraint the oldest occupations and drawings to between 37 and 34 ka cal BP [5]. The Chauvet-Pont d’Arc bestiary is particularly renowned for the predominance of so-called "dangerous animals" (e.g. cave lions, mammoths, rhinoceros), which are rather uncommon in Upper Palaeolithic iconography from Western Europe [7]. This bestiary also contains more classical animal drawings (e.g. horse, bison, megaloceros, ibex…) and human representations (negative and positive hands, vulvas, female lower body). This figurative bestiary coexists with a wide range of engraved or painted abstract signs (schematic “W”, "butterfly" and “spray-shape”) some of them unique to the Chauvet-Pont d’Arc cave [7,8]. The meaning of some of these signs is still unknown or subject to several hypotheses including, as we suggest here, depictions of volcanic eruptions. Hereafter we examine the possible meaning of the spray-shape signs (Fig 1C) in the light of new ^40^Ar/^39^Ar ages obtained on the Bas-Vivarais strombolian volcanoes located at 35 km northwest of the Chauvet-Pont d’Arc cave (Fig 1B). Indeed, the strombolian cones are visible from the hills located only few kilometers away from the cave entrance (Fig 1D).

## The Bas-Vivarais Volcanism

The Bas-Vivarais volcanic field (Southeast end of the French Massif Central) is dispersed over 500 km² [9] and constituted by seventeen eruptive centers aligned along NW-SE faults (Fig 1B). This French region is an iconic place for volcanologists because in 1778 the geologist Faujas de Saint-Font has linked for the first time prismatic basaltic flows and volcanic activity at the Coupe d’Aizac cone [10,11]. Since 2014, the Bas-Vivarais region that evidences 500 Ma of the Earth history is recognized as World Geopark by United Nations Educational, Scientific and Cultural Organization. The Bas-Vivarais volcanism is characterized by phreatomagmatic eruptions (maars) preceded or followed by strombolian activity associated with the emission of lava flows filling pre-exiting valleys [12] (Fig 1B). The volcanic products are mainly alkali basalts derived from a unique enriched mantle source [13,14]. This activity has always been considered as one of the most recent in the entire Massif Central and suggested to be as young as the Chaîne des Puys volcanic field [10,11,15] (Fig 1A). Despite several attempts, the chronological framework of this volcanic activity remains poorly constrained. Only eight volcanic centers have been dated by thermoluminescence and ^14^C [12,15]. The volcanic activity is currently divided into three distinct phases. The oldest one, developed at the north of the area, in the high plateau, predates the last interglacial with a mean age of 166 ± 15 ka [12]. South of the studied area, more recent eruptive centers, associated with the valley filling lava flows, are found (Fig 1). The ages corresponding to this recent activity are clustered into two age groups respectively centered at 79 ± 5 ka and 45 ± 3 ka [12]. Because of the frequent occurrences of mantle and lower crust xenoliths in the lavas [12] no radiometric age determination of the recent activity is currently available. To improve the chronology of this young volcanic activity a study [16] has provided the first ^40^Ar/^39^Ar ages on the youngest activity of the Bas-Vivarais. We will present for the first time below some of these new radio-isotopic constraints.

## Material and Methods

All samples presented hereafter (i.e. *Suc de Bauzon*, *Coupe d’Aizac*, and *le maar de Ray-Pic)* were collected in June 2012, two years before the UNESCO labeled the Bas-Vivarais as World Geopark. Therefore, at the time, no specific permission was required for all the locations we investigated. Moreover, the field study did not involve endangered or protected species.

**Fig 2:**
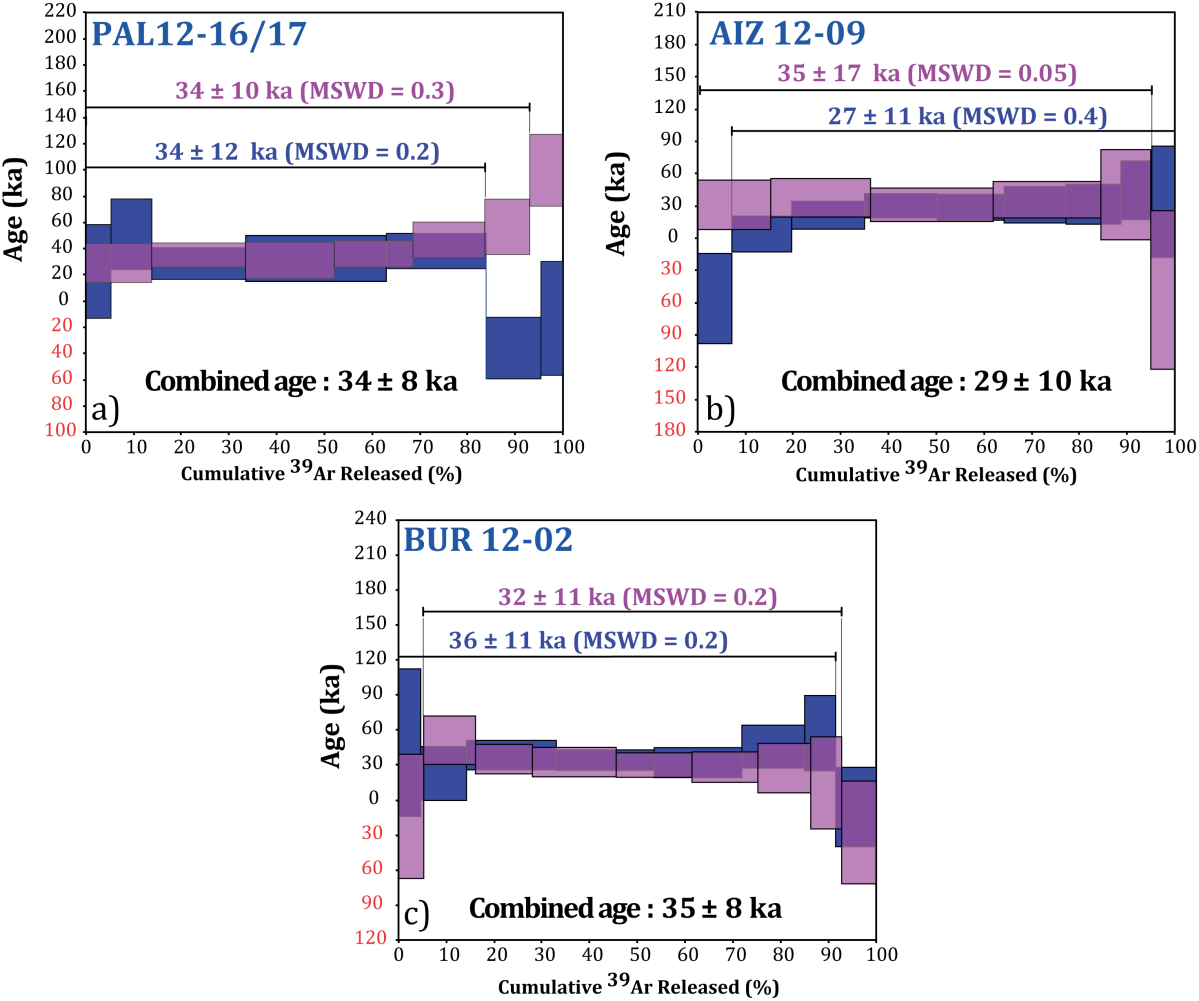
Plateau spectra for the three volcanic centers dated. Uncertainties are given at the 2σ level. Plateau steps are plotted at the 1σ level.

^40^Ar/^39^Ar ages were obtained by step-heating experiments on groundmass (120 to 150 mg) at the LSCE argon facility (France) using a high-sensitivity noble gas GV5400 instrument operated in ion counting mode. Irradiation procedures, extraction and gas cleanup, mass spectrometric measurements and blank corrections, are fully described in [17]. Ages are calculated using ACs-2 standard at 1.193 ± 0.01 Ma [18] and the total decay constant of Steiger and Jäger [19]. Several proposed calibrations of the ^40^Ar/^39^Ar chronometer are currently in use, yielding ages that vary by ~1% [20,21]. However, this implied difference in calibrated age of 250 to 300 years; thus negligible compared to the individual plateau age uncertainty (*e*.*g*. 11 ka, Fig 2). Correction factors for interfering neutron reactions were determined on pure compounds (K_2_O, CaF_2_) irradiated in the same position and were: (^39^Ar/^37^Ar) _Ca_ = 8.050 10^−4^, (^36^Ar/^37^Ar) _Ca_ = 3.765 10^−4^, (^40^Ar/^39^Ar) _K_ = 6.560 10^−4^; (^38^Ar/^39^Ar) _K_ = 1.120 10^−2^. Mass discrimination was assessed by analysis of Air pipette throughout the analytical period, and was calculated relative to a ^40^Ar/^36^Ar ratio of 298.56 [22]. ^40^Ar/^39^Ar full analytical dataset and furnace blanks are provided as supporting material (S1 and S2 Tables).

## Results

### ^40^Ar/^39^Ar constraints on the Bas-Vivarais youngest volcanic activity

We present hereafter, ages obtained on three volcanic centers. They are easily visible from the Chauvet-Pont d’Arc cave vicinity and named *le Suc du Bauzon* (PAL 12-16-17); *Coupe d’Aizac* (AIZ 12–09); and *maar de Ray-Pic* (BUR 12–02) (Fig 1B). Step heating experiments provided flat spectra including 87.4 to 95.0% of the total extracted gas and therefore defined valid age plateaus (Fig 2). The ^40^Ar* contents of the plateau steps are ranging between 1% and 3%. Inverse isochron diagrams do not evidence apparent excess argon. Initial ^40^Ar/^36^Ar ratios are identical within uncertainty with the modern ^40^Ar/^36^Ar atmospheric ratio (S1 and S2 Tables). However, the spreading along them is very limited, lower than 2%, resulting on large uncertainties for the inverse isochron ages. Therefore, the plateau ages are preferred and retrained to date these eruptions. All plateau ages are clustered between 36 ± 11 ka and 27 ± 11 ka (2σ) (Fig 2 and S1 Table). Age reported hereafter for each volcanic center is the combined age of the two step-heating experiments and given at the 2σ level (Fig 2).

### The spray-shape signs in the Chauvet-Pont d’Arc cave

**Fig 3:**
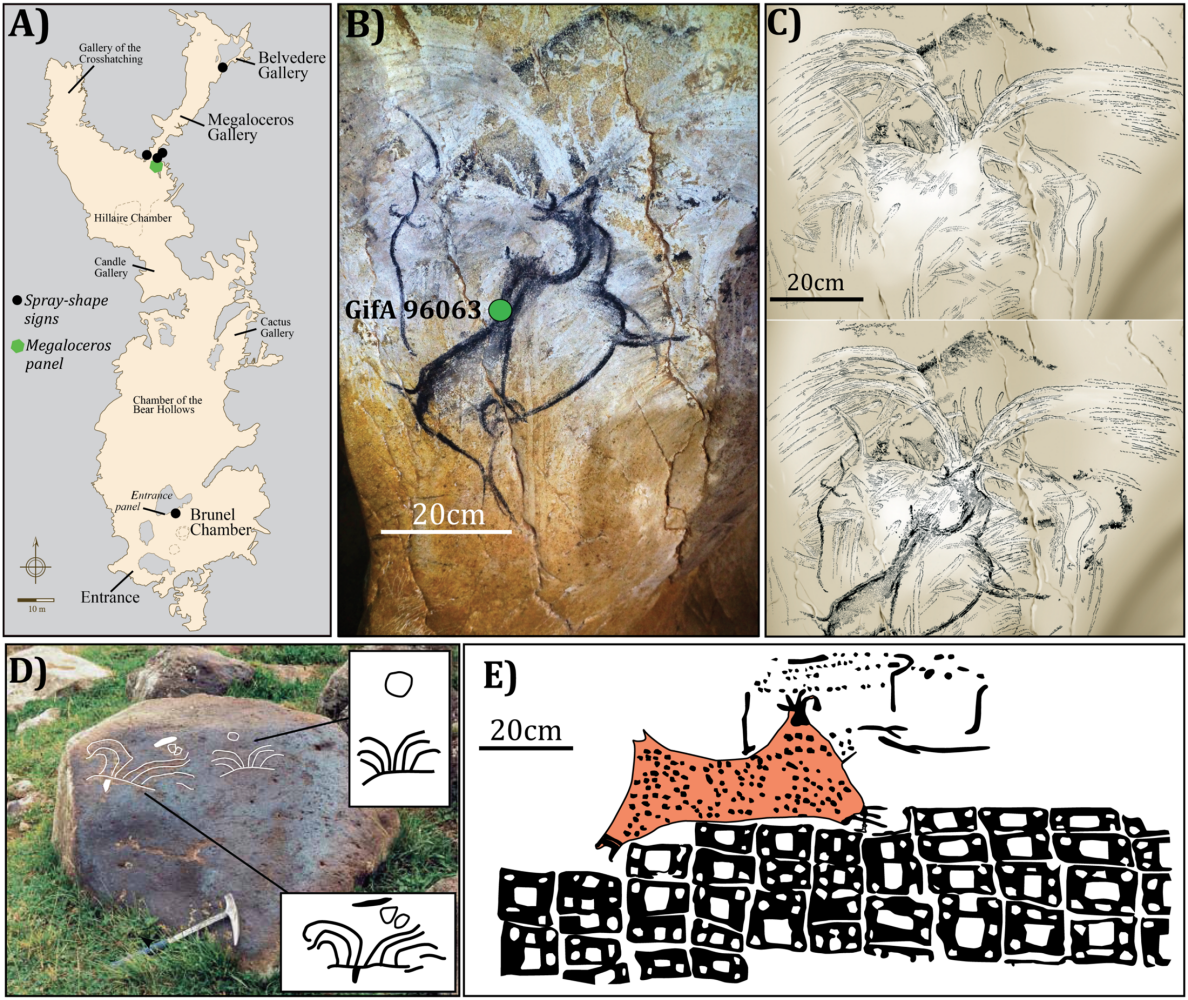
Example of a spray-shape sign from Chauvet-Pont d’Arc cave compared to the oldest known depictions of volcanic eruptions. (A) Map of the Chauvet-Pont d’Arc cave. (B) General view of the Megaloceros panel. The green dot marks the location of the ^14^C AMS date [8] (picture credit D. Genty). (C) Detail of the Megaloceros panel chronological succession [8] (pictures credit V. Feruglio-D. Baffier). (D) Petroglyphs depicting the Porak volcano eruption and dated from the 5^th^ millennium BC in the Syunik region of Armenia [3]. The figure is similar to [3] but not identical to the original image, and is therefore for illustrative purposes only. (E) Çatalhöyük mural painting (Turkey) considered the oldest depiction of a volcanic eruption dated from the 8^th^/7^th^ millennium BC [1].

These signs consist of two diverging arrays of curved lines [8] (Figs 1C–3B and 3C). Four occurrences were found in the Megaloceros and Belvedere Galleries and one near the original entrance of the cave in the Brunel Chamber [8] (Fig 3A). These signs were either traced using fingers in the Megaloceros and Belvedere Galleries or painted with red pigment like that found in the Entrance panel in the Brunel chamber [8] (Fig 3A). The chronological succession of the Megaloceros panel superposition described in Féruglio and Baffier [8] is complex. Four phases can be separated (Fig 3C):

1. First phase of drawings.
2. Cave bear marks.
3. Spray-shape sign engraving.
4. The spray-shape sign is partially covered by a Megaloceros drawing made using charcoal and with a stumping technique (which is not the case of the figures of the phase 1).

### Chronological relationship between the spray-shape signs and the eruption ages

**Fig 4:**
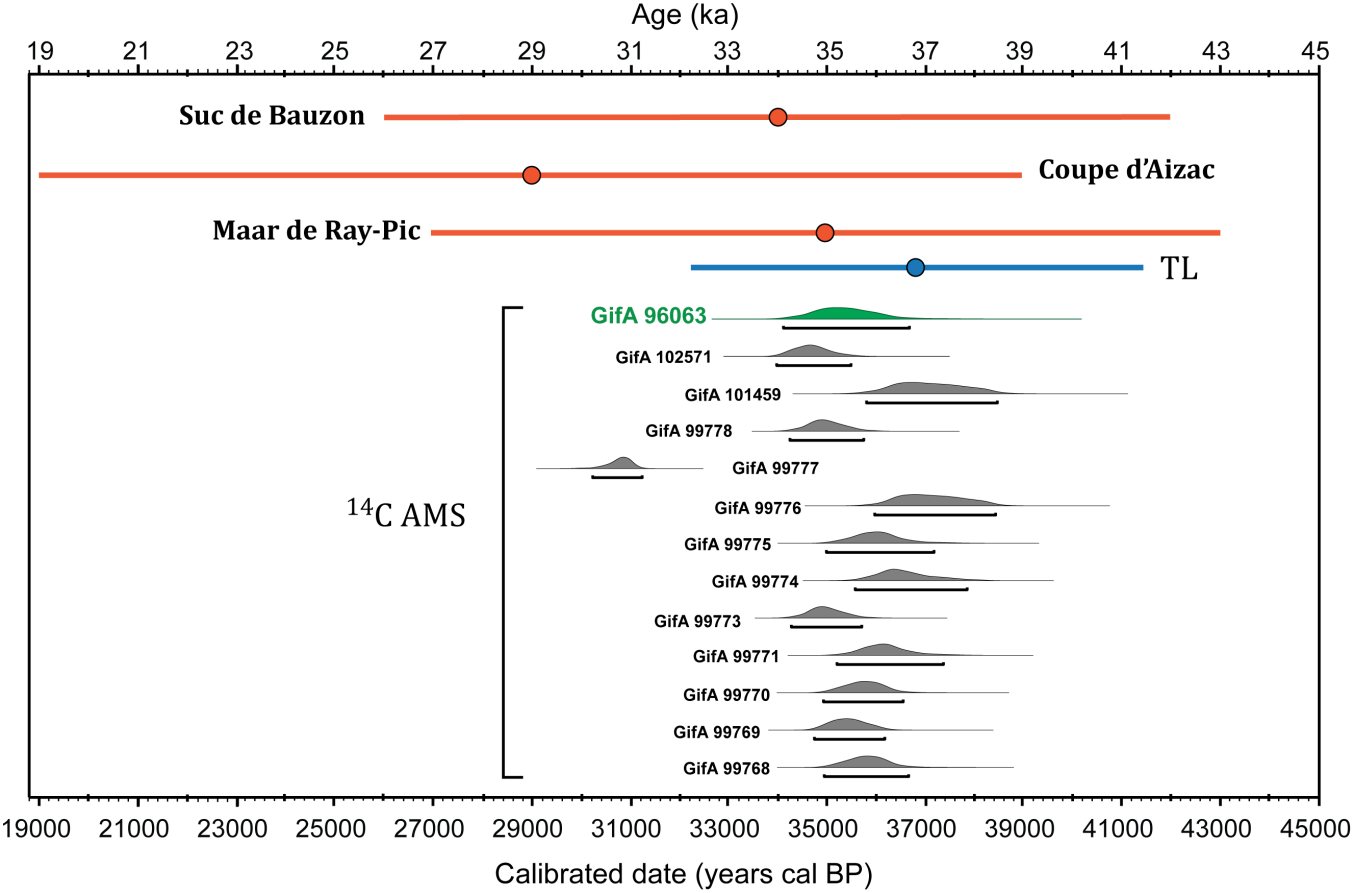
Volcanic centers ^40^Ar/^39^Ar ages and spray-shape sign TL and ^14^C AMS dates. In red: ^40^Ar/^39^Ar ages of the three volcanic centers studied; blue: TL age on reddened limestone in the Megaloceros Gallery [26]; ^14^C AMS dates correspond to the first occupation of the Megaloceros Gallery in the Chauvet-Pont d’Arc cave [5]. The ^14^C AMS in green corresponds to the date obtained for the sample taken from the rump of the Megaloceros [8]. Uncertainties are reported at the 2σ level excluding TL age where uncertainty is an estimated standard deviation [26].

The chronology of the Chauvet-Pont d’Arc cave is currently based on numerous ^14^C AMS analyses of charcoal pigments as well as of charcoal fragments from hearths and bones [5,23,24]. These ^14^C AMS dates are consistent with U/Th ages on speleothems [25] as well as by recent TL age determinations of reddened limestone [26]. The current chronology defines two distinct phases of human presence, one at 37–34 ka cal BP and the other between 31 and 29 ka cal BP [5,24]. The recent ^14^C cross-comparison program for the Chauvet-Pont d’Arc cave points to several periods of occupation in the Megaloceros Gallery itself within a short period between 37 and 36 ka cal BP [5] (Fig 4). These chronological constraints are in agreement with the Megaloceros panel drawings superposition that suggests, at least three phases of ornamentation separated in one instant by the presence of cave bears [8]. One radiocarbon date is particularly relevant to constrain the age of the spray-shape signs discovered in the Chauvet-Pont d’Arc cave. This date was obtained from charcoal pigments collected from the rump of the Megaloceros drawing that overlaps and covers the spray-shape sign (Figs 3 and 4), implying than the sign is older than 36.7 and 34.1 ka cal BP (Intcal 13) [24], gives us a minimum age for the spray-shape sign. Furthermore, another one of the spray-shape sign found in the Megaloceros Gallery is affected by cracks due to fires [26]. Thermoluminescence dating constraint the age of the fires in this gallery to 36.9 ± 2.3 ka [26], therefore, predating the Gravettian period. Our ^40^Ar/^39^Ar eruption ages, obtained from volcanoes easily visible from the cave vicinity, are contemporaneous with the Aurignacian cultural occupation of the cave, particularly with the ornamentations of the Megaloceros Gallery (Fig 4).

The distinctive design of spray-shape signs specific to Chauvet-Pont d’Arc cave is reminiscence of typical lava fountains associated with strombolian eruptions. Such symbols have no close equivalent within the considered time period (*i*.*e* Aurignacian), or even among the more than 340 known ornate caves in France and Spain. Additionally, the iconography of these signs closely matches that of petroglyphs interpreted as depicting the Porak volcano eruption [3] (Fig 3). The uncertainties on our ^40^Ar/^39^Ar ages preclude temporal correlations at sub-millennial scales between these signs and specific eruptions. Nevertheless, we present strong evidence that the volcanic activity in the Bas-Vivarais was coeval with Aurignacian occupation. Therefore the hypothesis that this spray shape signs represent volcanic eruptions must be considered probable and warrant further investigations. The observation that drawing these signs erased preexisting animal figures might arguably be interpreted as expressing the power of the volcanic event.

Finally, the volcano hypothesis should also be considered in light of the Aurignacians choice of animal iconography (*i*.*e*. mostly dangerous and powerful animals such as cave Lions, mammoths and Rhinoceros), all of which were probably not hunted by humans. We must add that as there is no other example in prehistoric rock art of natural scenery it is here perhaps the strength of the eruption image that might have inspired the Aurignacian artists. But as the rendering of animal figures is not fully realistic, those signs remain symbolic (the volcano itself is not drawn for instance). The Chauvet-Pont d’Arc is the cave of the artistic exceptions in terms of technic, themes, composition and visual innovations for the time period considered. It will not be surprising to find the first depiction of a volcanic eruption in human history among the ornmentations of this exceptional cave.

## Conclusion

New ^40^Ar/^39^Ar constraints in the Bas-Vivarais (Ardèche, France) provides radiometric evidence for a volcanic activity between 30 and 40 ka, coeval with the Aurignacian occupation of the Chauvet-Pont d’Arc cave, only 35 km southwest. Spray-shape signs specific to this cave art are dated between 36.7 and 34.1 ka cal BP. They overlapping the age of the local volcanic activity. We propose that humans are likely to have witnessed one or several eruptions and depicted them using these complex signs. If this hypothesis is correct, these depictions predate over 34 millennia the observation by Pliny the Younger of the AD 79 Vesuvius eruption and by 28 ka the Çatalhöyük mural (Turkey), currently considered the oldest eruption painted by a human hand.

## Supporting Information

S1 Table ^40^Ar/^39^Ar full dataset. Data are corrected from the blanks and discrimination. Data in red are not used to calculate plateau or inverse isochron ages.
(PDF)

S2 Table ^40^Ar/^39^Ar blanks dataset. (PDF)
