# Supplementary material for: A 36,000-Year-Old Volcanic Eruption Depicted in the Chauvet-Pont d’Arc Cave (Ardèche, France)?
Source: PLoS One. 2016 Jan 8;11(1):e0146621. doi: 10.1371/journal.pone.0146621 (PMC4706433; doi:10.1371/journal.pone.0146621)
Supplement: S1 Table — Data are corrected from the blanks and discrimination. Data in red are not used to calculate plateau or inverse isochron ages. (PDF) [file pone.0146621.s001.pdf]

BUR12-02A<br>Analyse N°	124 mg<br>Temperature	36Ar<br>[V]	%1σ	37Ar<br>[V]	%1σ	38Ar<br>[V]	%1σ	39Ar<br>[V]	%1σ	40Ar<br>[V]	%1σ	40(r)/39(k)	± 2σ	Age	± 2σ<br>(Ka)	40Ar(r)<br>(%)	39Ar(k)<br>(%)	K/Ca	± 2σ
FG-955	650 °C	3,049E-05	4,182E-01	1,613E-04	8,947E-01	1,920E-05	5,741E-01	5,877E-04	2,417E-01	9,068E-03	6,805E-02	0,02771	± 0,13126	23,1	± 109,3	0.18	5,33	0,547	± 0,010
FG-956	708 °C	2,480E-05	3,993E-01	3,324E-04	5,544E-01	3,478E-05	3,522E-01	1,181E-03	1,513E-01	7,438E-03	6,590E-02	0,06146	± 0,05075	51,2	± 42,3	0,98	10,71	0,533	± 0,006
FG-957	761 °C	1,501E-05	4,361E-01	3,417E-04	5,744E-01	3,874E-05	3,906E-01	1,326E-03	1,720E-01	4,499E-03	1,017E-01	0,04217	± 0,03030	35,1	± 25,2	1,24	12,02	0,582	± 0,007
FG-958	830 °C	1,716E-05	5,612E-01	4,781E-04	5,669E-01	5,469E-05	3,104E-01	1,932E-03	1,347E-01	5,147E-03	9,680E-02	0,03970	± 0,03023	33,1	± 25,2	1,49	17,51	0,606	± 0,007
FG-959	882 °C	1,646E-05	4,515E-01	5,172E-04	5,668E-01	4,743E-05	2,876E-01	1,757E-03	1,452E-01	4,920E-03	8,602E-02	0,03636	± 0,02574	30,3	± 21,4	1,30	15,92	0,509	± 0,006
FG-960	942 °C	1,875E-05	4,175E-01	5,320E-04	4,755E-01	3,875E-05	3,791E-01	1,523E-03	1,547E-01	5,591E-03	9,194E-02	0,03441	± 0,03144	28,7	± 26,2	0,94	13,80	0,429	± 0,004
FG-961	1002 °C	2,245E-05	4,598E-01	5,217E-04	4,834E-01	3,053E-05	4,434E-01	1,220E-03	1,983E-01	6,684E-03	6,163E-02	0,03291	± 0,05100	27,4	± 42,5	0,60	11,05	0,351	± 0,004
FG-962	1060 °C	2,290E-05	4,882E-01	3,809E-04	5,195E-01	1,930E-05	5,209E-01	7,091E-04	1,923E-01	6,807E-03	7,657E-02	0,01808	± 0,09532	15,1	± 79,4	0,19	6,43	0,279	± 0,003
FG-963	1154 °C	2,944E-05	4,794E-01	5,618E-04	5,177E-01	2,071E-05	4,030E-01	7,994E-04	1,640E-01	8,701E-03	5,976E-02	0,03345	± 0,10631	27,9	± 88,5	0,31	7,24	0,213	± 0,002

J = 0.00045500 ± 0.00000378
ACS-2 = 1.193 ± 0.001 Ma
Age Equations = Min et al. (2000)
Irradiation = IRR 73
Mass Discrimination Law = Power law
Scaling Ratio K/Ca = 0.150

Results	40(a)/36(a) ± 2σ	40(r)/39(k) ± 2σ	Age ± 2σ (ka)	MSWD	39Ar(k) (%,n)	K/Ca ± 2σ
Age Plateau		0.03894 0.01338 34.4%	32,4 ± 11,2 34.4%	0.20	87.4	0.399 0.091
Inverse Isochron	298.75 2.95 1.0%		30.5 27.5 90.7%	0.24	87.4	

BUR12-02B<br>Analyse N°	144 mg<br>Temperature	36Ar<br>[V]	%1σ	37Ar<br>[V]	%1σ	38Ar<br>[V]	%1σ	39Ar<br>[V]	%1σ	40Ar<br>[V]	%1σ	40(r)/39(k)	± 2σ	Age	± 2σ<br>(Ka)	40Ar(r)<br>(%)	39Ar(k)<br>(%)	K/Ca	± 2σ
FG-974	650 °C	3,659E-05	4,282E-01	1,678E-04	3,398E+00	2,166E-05	3,197E-01	6,532E-04	2,029E-01	1,094E-02	0,117	0,05752	± 0,14854	49,8	± 128,7	0,34	4,56	0,584	± 0,040
FG-975	723 °C	3,030E-05	3,811E-01	3,880E-04	1,558E+00	3,996E-05	3,457E-01	1,386E-03	1,477E-01	9,040E-03	0,158	0,02659	± 0,05387	23,0	± 46,7	0,41	9,67	0,536	± 0,017
FG-976	799 °C	2,537E-05	5,019E-01	6,893E-04	9,121E-01	7,673E-05	3,061E-01	2,710E-03	1,875E-01	7,618E-03	0,164	0,04546	± 0,02955	39,4	± 25,6	1,62	18,91	0,590	± 0,011
FG-977	860 °C	2,429E-05	3,894E-01	7,959E-04	8,304E-01	8,041E-05	2,460E-01	2,911E-03	1,063E-01	7,279E-03	0,117	0,04028	± 0,02028	34,9	± 17,6	1,61	20,32	0,549	± 0,009
FG-978	930 °C	3,053E-05	4,179E-01	9,085E-04	7,528E-01	6,704E-05	1,449E-01	2,650E-03	1,140E-01	9,113E-03	0,113	0,03750	± 0,02982	32,5	± 25,8	1,09	18,49	0,437	± 0,007
FG-979	996 °C	3,140E-05	4,239E-01	7,595E-04	8,434E-01	4,655E-05	3,817E-01	1,889E-03	1,221E-01	9,389E-03	0,096	0,05333	± 0,04318	46,2	± 37,4	1,07	13,18	0,373	± 0,006
FG-980	1055 °C	2,669E-05	4,165E-01	4,389E-04	1,334E+00	2,458E-05	2,162E-01	9,248E-04	1,480E-01	7,981E-03	0,135	0,06720	± 0,07550	58,2	± 65,4	0,78	6,45	0,316	± 0,008
FG-981	1155 °C	4,529E-05	3,462E-01	7,625E-04	8,545E-01	3,211E-05	3,402E-01	1,205E-03	2,122E-01	1,343E-02	0,085	0,00761	± 0,07999	6,6	± 69,3	0,07	8,41	0,237	± 0,004

J = 0.00047350 ± 0.00000327
ACS-2 = 1.193 ± 0.001 Ma
Age Equations = Min et al. (2000)
Irradiation = IRR 73
Mass Discrimination Law = Power law
Scaling Ratio K/Ca = 0.150

Results	40(a)/36(a) ± 2σ	40(r)/39(k) ± 2σ	Age ± 2σ (ka)	MSWD	39Ar(k) (%,n)	K/Ca ± 2σ
Age Plateau		0,04210 ± 0,01313 31.2%	36.5 11.4 31.2%	0.21 n//N=	91.6 7//8	0.434 0.074
Inverse Isochron	299.0 2.1 0.7%		32.5 22.5 69.3%	0.22		

AIZ12-09A<br>Analyse N°	150 mg<br>Temperature	36Ar<br>[V]	%1σ	37Ar<br>[V]	%1σ	38Ar<br>[V]	%1σ	39Ar<br>[V]	%1σ	40Ar<br>[V]	%1σ	40(r)/39(k)	± 2σ	Age	± 2σ<br>(Ka)	40Ar(r)<br>(%)	39Ar(k)<br>(%)	K/Ca	± 2σ
FG-946	650 °C	1,686E-05	5,237E-01	4,624E-04	5,744E-01	1,634E-05	5,270E-01	5,320E-04	2,029E-01	4,945E-03	1,441E-01	0,06731	± 0,10275	55,2	± 84,3	0,72	7,12	0,172	± 0,002
FG-947	701 °C	1,156E-05	5,379E-01	7,109E-04	5,131E-01	2,574E-05	3,825E-01	9,397E-04	1,097E-01	3,377E-03	1,147E-01	0,00576	± 0,04044	4,7	± 33,2	0,16	12,58	0,198	± 0,002
FG-948	752 °C	8,038E-06	7,302E-01	8,000E-04	4,688E-01	2,908E-05	4,189E-01	1,144E-03	1,664E-01	2,341E-03	1,428E-01	0,02711	± 0,03124	22,2	± 25,6	1,32	15,32	0,214	± 0,002
FG-949	809 °C	7,274E-06	6,903E-01	8,294E-04	4,636E-01	2,732E-05	4,149E-01	1,137E-03	2,551E-01	2,122E-03	1,725E-01	0,03798	± 0,02721	31,1	± 22,3	2,03	15,22	0,206	± 0,002
FG-950	865 °C	7,594E-06	6,263E-01	8,498E-04	4,443E-01	2,048E-05	5,431E-01	1,043E-03	2,516E-01	2,209E-03	2,046E-01	0,03547	± 0,02865	29,1	± 23,5	1,67	13,96	0,184	± 0,002
FG-951	912 °C	7,378E-06	9,031E-01	8,316E-04	4,969E-01	1,637E-05	5,995E-01	9,758E-04	1,763E-01	2,147E-03	1,476E-01	0,03876	± 0,04136	31,8	± 33,9	1,76	13,06	0,176	± 0,002
FG-952	966 °C	7,380E-06	8,797E-01	9,070E-04	4,716E-01	1,403E-05	5,483E-01	8,628E-04	2,539E-01	2,136E-03	1,440E-01	0,04033	± 0,04560	33,1	± 37,4	1,63	11,55	0,143	± 0,002
FG-953	1026 °C	7,484E-06	6,807E-01	7,150E-04	5,384E-01	8,807E-06	1,057E+00	4,795E-04	2,408E-01	2,181E-03	2,238E-01	0,05603	± 0,06680	46,0	± 54,8	1,23	6,42	0,100	± 0,001
FG-954	1104 °C	1,309E-05	5,509E-01	7,828E-04	4,732E-01	8,733E-06	7,993E-01	3,572E-04	2,814E-01	3,837E-03	1,629E-01	0,04455	± 0,12588	36,5	± 103,2	0,41	4,78	0,068	± 0,001

J = 0.00044810 ± 0.00000269
ACS-2 = 1.193 ± 0.001 Ma
Age Equations = Min et al. (2000)
Irradiation = IRR 73
Mass Discrimination Law = Power law
Scaling Ratio K/Ca = 0.150

Results	40(a)/36(a) ± 2σ	40(r)/39(k) ± 2σ	Age ± éσ (ka)	MSWD	39Ar(k) (%,n)	K/Ca ± 2σ
Age Plateau		0.03297 ± 0,01341 40.68%	27.0 11.0 40.7%	0.40 n//N=	92.9 8//9	0.119 0.041
Inverse Isochron	298.33 3.6 1.2%		28.6 20.7 72.3%	0.46		

AIZ12-09B<br>Analyse N°	119 mg<br>Temperature	36Ar<br>[V]	%1σ	37Ar<br>[V]	%1σ	38Ar<br>[V]	%1σ	39Ar<br>[V]	%1σ	40Ar<br>[V]	%1σ	40(r)/39(k)	± 2σ	Age	± 2σ<br>(Ka)	40Ar(r)<br>(%)	39Ar(k)<br>(%)	K/Ca	± 2σ
FG-1037	713 °C	3,012E-06	1,463E+00	3,189E-04	4,106E-01	1,159E-05	7,739E-01	4,405E-04	2,068E-01	8,858E-04	1,074E+00	0,05098	± 0,07378	31,7	± 45,9	2,53	15,36	0,207	± 0,002
FG-1038	784 °C	4,433E-06	1,087E+00	4,697E-04	3,895E-01	1,339E-05	5,311E-01	5,963E-04	1,808E-01	1,307E-03	7,220E-01	0,06071	± 0,05777	37,7	± 35,9	2,77	20,79	0,190	± 0,002
FG-1039	840 °C	5,735E-06	9,424E-01	6,023E-04	3,802E-01	1,336E-05	4,517E-01	7,422E-04	1,723E-01	1,683E-03	5,595E-01	0,05097	± 0,05041	31,7	± 31,3	2,25	25,88	0,185	± 0,002
FG-1040	900 °C	5,892E-06	8,362E-01	6,844E-04	3,759E-01	1,180E-05	4,340E-01	6,446E-04	1,807E-01	1,720E-03	5,512E-01	0,05791	± 0,05438	36,0	± 33,8	2,17	22,47	0,141	± 0,001
FG-1041	960 °C	6,504E-06	9,115E-01	4,759E-04	3,801E-01	6,408E-06	1,072E+00	3,015E-04	3,844E-01	1,908E-03	5,211E-01	0,06583	± 0,13490	40,9	± 83,8	1,04	10,50	0,095	± 0,001
FG-1042	1017 °C	6,126E-06	7,919E-01	3,106E-04	3,896E-01	4,034E-06	1,269E+00	1,436E-04	5,041E-01	1,782E-03	5,559E-01	0,08350	± 0,24488	51,9	± 152,2	0,67	5,00	0,069	± 0,001

J = 0.00033960 ± 0.00000228
ACS-2 = 1.193 ± 0.001 Ma
Age Equations = Min et al. (2000)
Irradiation = IRR 79
Mass Discrimination Law = Power law
Scaling Ratio K/Ca = 0.150

Results	40(a)/36(a) ± 2σ	40(r)/39(k) ± 2σ	Age ± 2σ (ka)	MSWD	39Ar(k) (%,n)	K/Ca ± 2σ
Age Plateau		0.05576 ± 0,02806 50.3%	34.7 17.4 50.3%	0.03 n//N=	95.0 5//6	0.145 0.042
Inverse Isochron	299.4 9.9 3."%		30.2 51.8 171.25%	0.03		

PIC12-08<br>Analyse N°	143 mg<br>Temperature	36Ar<br>[V]	%1σ	37Ar<br>[V]	%1σ	38Ar<br>[V]	%1σ	39Ar<br>[V]	%1σ	40Ar<br>[V]	%1σ	40(r)/39(k)	± 2σ	Age	± 2σ<br>(Ka)	40Ar(r)<br>(%)	39Ar(k)<br>(%)	K/Ca	± 2σ
FG-876	594 °C	8,639E-06	7,087E-01	1,548E-04	8,262E-01	7,493E-06	9,520E-01	2,435E-04	4,153E-01	2,551E-03	2,297E-01	0,04391	± 0,15778	36,6	± 131,6	0,42	2,77	0,236	± 0,004
FG-877	647 °C	5,570E-06	1,259E+00	2,055E-04	6,006E-01	9,417E-06	1,087E+00	3,337E-04	4,367E-01	1,609E-03	4,502E-01	0,09301	± 0,13285	77,6	± 110,8	1,93	3,80	0,243	± 0,004
FG-878	715 °C	6,529E-06	9,295E-01	5,038E-04	4,605E-01	2,400E-05	3,723E-01	8,597E-04	1,731E-01	1,960E-03	2,907E-01	0,07860	± 0,04423	65,5	± 36,9	3,45	9,78	0,256	± 0,003
FG-879	800 °C	8,326E-06	1,225E+00	9,796E-04	3,999E-01	4,479E-05	3,643E-01	1,874E-03	2,063E-01	2,470E-03	2,464E-01	0,05058	± 0,03318	42,2	± 27,7	3,84	21,32	0,287	± 0,003
FG-880	883 °C	1,078E-05	5,375E-01	1,284E-03	3,958E-01	4,084E-05	3,651E-01	2,216E-03	2,063E-01	3,147E-03	2,108E-01	0,03321	± 0,01678	27,7	± 14,0	2,34	25,21	0,259	± 0,002
FG-881	961 °C	1,368E-05	5,639E-01	1,623E-03	3,688E-01	3,511E-05	3,003E-01	2,123E-03	1,433E-01	3,970E-03	1,635E-01	0,03158	± 0,02262	26,3	± 18,9	1,69	24,15	0,196	± 0,002
FG-882	1026 °C	9,255E-06	8,502E-01	8,400E-04	3,973E-01	1,325E-05	5,099E-01	7,357E-04	1,992E-01	2,684E-03	2,022E-01	0,01967	± 0,06566	16,4	± 54,7	0,54	8,37	0,131	± 0,001
FG-883	1103 °C	1,234E-05	5,748E-01	7,085E-04	4,029E-01	9,030E-06	6,796E-01	4,050E-04	2,829E-01	3,559E-03	1,681E-01	0,10933	± 0,10884	91,2	± 90,8	1,24	4,60	0,086	± 0,001

J = 0.00045560 ± 0.00000524
ACS-2 = 1.193 ± 0.001 Ma
Age Equations = Min et al. (2000)
Irradiation = IRR 72
Mass Discrimination Law = Power law
Scaling Ratio K/Ca = 0.150

Results	40(a)/36(a) ± 2σ	40(r)/39(k) ± 2σ	Age ± 2σ (ka)	MSWD	39Ar(k) (%,n)	K/Ca ± 2σ
Age Plateau		0.03770 ± 0,01309 34.7%	31.5 10.9 34.8%	1.23 n//N=	88.8 5//8	0.188 0.058
Inverse Isochron	298.8 9.7 3.25%		29.8 27.3 91.7%	1.64		

PAL12-16<br>Analyse N°	121 mg<br>Temperature	36Ar<br>[V]	%1σ	37Ar<br>[V]	%1σ	38Ar<br>[V]	%1σ	39Ar<br>[V]	%1σ	40Ar<br>[V]	%1σ	40(r)/39(k)	± 2σ	Age	± 2σ<br>(Ka)	40Ar(r)<br>(%)	39Ar(k)<br>(%)	K/Ca	± 2σ
FG-1070	652 °C	8,565E-06	6,500E-01	2,318E-04	4,067E-01	1,422E-05	5,358E-01	5,533E-04	1,226E-01	2,562E-03	2,511E-01	0,05556	± 0,06446	25,7	± 29,8	1,20	14,04	0,358	± 0,003
FG-1071	707 °C	7,088E-06	6,530E-01	2,790E-04	3,367E-01	1,796E-05	5,311E-01	7,658E-04	1,840E-01	2,137E-03	2,836E-01	0,06846	± 0,03943	31,7	± 18,2	2,45	19,43	0,412	± 0,003
FG-1072	757 °C	6,435E-06	1,111E+00	2,751E-04	3,660E-01	1,616E-05	6,698E-01	7,387E-04	1,841E-01	1,934E-03	3,158E-01	0,05933	± 0,06012	27,5	± 27,8	2,26	18,74	0,403	± 0,003
FG-1073	811 °C	6,169E-06	7,047E-01	2,938E-04	3,925E-01	1,259E-05	4,817E-01	6,497E-04	1,841E-01	1,856E-03	3,055E-01	0,07322	± 0,04363	33,9	± 20,2	2,56	16,49	0,332	± 0,003
FG-1074	877 °C	6,799E-06	8,015E-01	3,695E-04	3,478E-01	1,093E-05	7,497E-01	5,880E-04	1,481E-01	2,044E-03	2,814E-01	0,09461	± 0,05875	43,8	± 27,2	2,72	14,92	0,239	± 0,002
FG-1075	944 °C	6,798E-06	7,757E-01	4,086E-04	3,264E-01	7,577E-06	5,736E-01	3,699E-04	2,699E-01	2,022E-03	2,969E-01	0,10453	± 0,09123	48,4	± 42,2	1,91	9,38	0,136	± 0,001
FG-1076	1017 °C	0,0000092	0,557	0,0004596	0,336	0,0000071	0,909	0,0002762	0,316	0,0027525	0,218	0,21817	± 0,11910	101,0	± 55,1	2,19	7,00	0,090	± 0,001

J = 0.00025290 ± 0.00000202
ACS-2 = 1.193 ± 0.001 Ma
Age Equations = Min et al. (2000)
Irradiation = IRR 80
Mass Discrimination Law = Power law
Scaling Ratio K/Ca = 0.150

Results	40(a)/36(a) ± 2σ	40(r)/39(k) ± 2σ	Age ± 2σ (ka)	MSWD	39Ar(k) (%,n)	K/Ca ± 2σ
Age Plateau		0.07265 ± 0,02184 30.1%	33.6 10.1 30.1%	0.31 n//N=	93.0 5//7	0.224 0.092
Inverse Isochron	300.2 8.1 2.7%		25.6 41.0 160.0%	0.35		

PAL12-17<br>Analyse N°	128 mg<br>Temperature	36Ar<br>[V]	%1σ	37Ar<br>[V]	%1σ	38Ar<br>[V]	%1σ	39Ar<br>[V]	%1σ	40Ar<br>[V]	%1σ	40(r)/39(k)	± 2σ	Age	± 2σ<br>(Ka)	40Ar(r)<br>(%)	39Ar(k)<br>(%)	K/Ca	± 2σ
FG-1028	656 °C	4,187E-06	1,032E+00	1,535E-04	7,101E+00	7,473E-06	1,121E+00	2,538E-04	3,766E-01	1,242E-03	4,592E-01	0,03566	± 0,11159	23,0	± 71,8	0,73	5,36	0,248	± 0,035
FG-1029	724 °C	4,966E-06	1,094E+00	2,945E-04	3,695E+00	1,319E-05	5,640E-01	4,003E-04	2,052E-01	1,481E-03	3,110E-01	0,07851	± 0,08452	50,6	± 54,4	2,12	8,45	0,204	± 0,015
FG-1030	786 °C	7,475E-06	7,567E-01	5,685E-04	1,924E+00	2,529E-05	3,211E-01	9,348E-04	2,282E-01	2,209E-03	2,303E-01	0,04454	± 0,03787	28,7	± 24,4	1,88	19,74	0,247	± 0,010
FG-1031	846 °C	8,309E-06	7,169E-01	7,168E-04	1,571E+00	3,075E-05	5,152E-01	1,398E-03	1,722E-01	2,471E-03	2,456E-01	0,05053	± 0,02698	32,5	± 17,4	2,86	29,53	0,292	± 0,009
FG-1033	965 °C	6,898E-06	9,751E-01	6,572E-04	1,721E+00	1,621E-05	6,937E-01	9,892E-04	1,853E-01	2,046E-03	2,338E-01	0,06044	± 0,04186	38,9	± 27,0	2,92	20,89	0,226	± 0,008
FG-1034	1025 °C	5,461E-06	1,149E+00	4,020E-04	2,784E+00	9,037E-06	1,009E+00	5,403E-04	2,571E-01	1,556E-03	3,566E-01	0,05493	± 0,07254	35,4	± 46,7	1,91	11,41	0,201	± 0,011
FG-1035	1080 °C	4,482E-06	1,114E+00	1,289E-04	8,424E+00	4,527E-06	1,201E+00	2,181E-04	5,372E-01	1,317E-03	3,704E-01	0,03014	± 0,14436	19,4	± 92,9	0,50	4,61	0,254	± 0,043

J = 0.00035180 ± 0.00000190
ACS-2 = 1.193 ± 0.001 Ma
Age Equations = Min et al. (2000)
Irradiation = IRR 74
Mass Discrimination Law = Power law
Scaling Ratio K/Ca = 0.150

Results	40(a)/36(a) ± 2σ	40(r)/39(k) ± 2σ	Age ± 2σ (ka)	MSWD	39Ar(k) (%,n)	K/Ca ± 2σ
Age Plateau		0.05200 ± 0,01869 35.9%	33.5 12.0 36.0%	0.20 n//N=	83.98 5//7	0.247 0.030
Inverse Isochron	298.9 8.7 2.9%		31.9 35.9 111.6%	0.27		
