# Supplementary material for: A 36,000-Year-Old Volcanic Eruption Depicted in the Chauvet-Pont d’Arc Cave (Ardèche, France)?
Source: PLoS One. 2016 Jan 8;11(1):e0146621. doi: 10.1371/journal.pone.0146621 (PMC4706433; doi:10.1371/journal.pone.0146621)
Supplement: S2 Table — (PDF) [file pone.0146621.s002.pdf]

BUR12-02A

Blanks		36Ar [V]	1σ	37Ar [V]	1σ	38Ar [V]	1σ	39Ar [V]	1σ	40Ar [V]	1σ
FG-956	708 °C	8,426E-07	2,482E-08	3,418E-07	7,491E-08	2,120E-07	1,940E-08	1,338E-07	8,498E-08	1,197E-04	2,389E-06
FG-957	761 °C	9,282E-07	2,482E-08	7,315E-07	7,410E-08	2,225E-07	1,940E-08	2,753E-07	8,498E-08	1,376E-04	2,363E-06
FG-958	830 °C	1,025E-06	2,482E-08	1,144E-06	7,338E-08	2,355E-07	1,940E-08	4,378E-07	8,498E-08	1,574E-04	2,340E-06
FG-959	882 °C	1,072E-06	2,482E-08	1,268E-06	7,307E-08	2,435E-07	1,940E-08	5,037E-07	8,498E-08	1,666E-04	2,330E-06
FG-960	942 °C	1,093E-06	2,482E-08	1,185E-06	7,298E-08	2,504E-07	1,940E-08	5,015E-07	8,498E-08	1,701E-04	2,327E-06
FG-961	1002 °C	1,083E-06	2,482E-08	9,356E-07	7,317E-08	2,555E-07	1,940E-08	4,250E-07	8,498E-08	1,676E-04	2,334E-06
FG-962	1060 °C	1,057E-06	2,482E-08	7,093E-07	7,363E-08	2,609E-07	1,940E-08	3,162E-07	8,498E-08	1,639E-04	2,348E-06
FG-963	1154 °C	1,045E-06	2,482E-08	1,094E-06	7,490E-08	2,790E-07	1,940E-08	2,330E-07	8,498E-08	1,725E-04	2,389E-06

BUR12-02B

Blanks		36Ar [V]	1σ	37Ar [V]	1σ	38Ar [V]	1σ	39Ar [V]	1σ	40Ar [V]	1σ
FG-974	650 °C	8,612E-07	2,709E-08	1,477E-06	5,419E-07	2,106E-07	2,173E-08	1,619E-07	8,413E-08	1,169E-04	6,365E-06
FG-975	723 °C	9,385E-07	2,709E-08	1,481E-06	5,313E-07	2,288E-07	2,173E-08	2,615E-07	8,413E-08	1,349E-04	6,241E-06
FG-976	799 °C	1,002E-06	2,709E-08	5,707E-07	5,227E-07	2,483E-07	2,173E-08	3,440E-07	8,413E-08	1,516E-04	6,140E-06
FG-977	860 °C	1,060E-06	2,709E-08	4,642E-08	5,178E-07	2,645E-07	2,173E-08	4,389E-07	8,413E-08	1,679E-04	6,082E-06
FG-978	930 °C	1,148E-06	2,709E-08	2,635E-07	5,143E-07	2,836E-07	2,173E-08	6,013E-07	8,413E-08	1,927E-04	6,041E-06
FG-979	996 °C	1,258E-06	2,709E-08	1,492E-06	5,131E-07	3,022E-07	2,173E-08	8,037E-07	8,413E-08	2,228E-04	6,027E-06
FG-980	1055 °C	1,377E-06	2,709E-08	3,361E-06	5,139E-07	3,189E-07	2,173E-08	1,004E-06	8,413E-08	2,548E-04	6,036E-06
FG-981	1155 °C	1,602E-06	2,709E-08	7,298E-06	5,189E-07	3,466E-07	2,173E-08	1,285E-06	8,413E-08	3,144E-04	6,096E-06

AIZ12-09A

Blanks		36Ar [V]	1σ	37Ar [V]	1σ	38Ar [V]	1σ	39Ar [V]	1σ	40Ar [V]	1σ
FG-946	6,500E+02	9,337E-07	3,061E-08	6,897E-07	1,566E-07	2,166E-07	2,482E-08	2,438E-07	6,677E-08	1,308E-04	2,128E-06
FG-947	7,010E+02	9,713E-07	3,061E-08	7,492E-07	1,545E-07	2,196E-07	2,482E-08	2,918E-07	6,677E-08	1,403E-04	2,100E-06
FG-948	7,520E+02	1,028E-06	3,061E-08	6,187E-07	1,528E-07	2,275E-07	2,482E-08	2,499E-07	6,677E-08	1,511E-04	2,076E-06
FG-949	8,090E+02	1,089E-06	3,061E-08	4,002E-07	1,513E-07	2,372E-07	2,482E-08	1,592E-07	6,677E-08	1,616E-04	2,056E-06
FG-950	8,650E+02	1,131E-06	3,061E-08	2,297E-07	1,503E-07	2,440E-07	2,482E-08	7,456E-08	6,677E-08	1,685E-04	2,043E-06
FG-951	9,120E+02	1,146E-06	3,061E-08	1,780E-07	1,499E-07	2,462E-07	2,482E-08	3,215E-08	6,677E-08	1,712E-04	2,037E-06
FG-952A	9,660E+02	1,141E-06	3,061E-08	2,502E-07	1,498E-07	2,448E-07	2,482E-08	2,985E-08	6,677E-08	1,716E-04	2,035E-06
FG-952B	1,026E+03	1,117E-06	3,061E-08	4,870E-07	1,502E-07	2,400E-07	2,482E-08	8,611E-08	6,677E-08	1,707E-04	2,041E-06
FG-954	1,104E+03	1,092E-06	3,061E-08	9,304E-07	1,516E-07	2,364E-07	2,482E-08	2,165E-07	6,677E-08	1,751E-04	2,060E-06

AIZ12-09B

Blanks		36Ar [V]	1σ	37Ar [V]	1σ	38Ar [V]	1σ	39Ar [V]	1σ	40Ar [V]	1σ
FG-1037	7,130E+02	1,438E-06	3,564E-08	1,270E-07	1,037E-08	3,195E-07	1,976E-08	1,212E-07	8,728E-08	3,018E-04	9,480E-06
FG-1038	7,840E+02	1,441E-06	3,515E-08	1,247E-07	1,023E-08	3,187E-07	1,976E-08	1,401E-07	8,728E-08	3,017E-04	9,349E-06
FG-1039	8,400E+02	1,477E-06	3,475E-08	1,263E-07	1,019E-08	3,236E-07	1,976E-08	1,442E-07	8,728E-08	3,075E-04	9,243E-06
FG-1040	9,000E+02	1,531E-06	3,443E-08	1,295E-07	1,019E-08	3,311E-07	1,976E-08	1,407E-07	8,728E-08	3,172E-04	9,157E-06
FG-1041	9,600E+02	1,592E-06	3,419E-08	1,325E-07	1,019E-08	3,392E-07	1,976E-08	1,345E-07	8,728E-08	3,296E-04	9,092E-06
FG-1042	1,017E+03	1,668E-06	3,400E-08	1,342E-07	1,019E-08	3,485E-07	1,976E-08	1,287E-07	8,728E-08	3,478E-04	9,042E-06

PIC12-18

Blanks		36Ar [V]	1σ	37Ar [V]	1σ	38Ar [V]	1σ	39Ar [V]	1σ	40Ar [V]	1σ
FG-876	5,940E+02	1,129E-06	4,082E-08	3,309E-07	1,526E-07	2,942E-07	2,196E-08	-4,756E-09	1,030E-07	1,304E-04	5,183E-06
FG-877	6,470E+02	1,147E-06	4,082E-08	6,013E-08	1,501E-07	2,865E-07	2,196E-08	1,520E-08	1,030E-07	1,461E-04	5,098E-06
FG-878	7,150E+02	1,160E-06	4,082E-08	1,030E-07	1,473E-07	2,789E-07	2,196E-08	8,027E-08	1,030E-07	1,528E-04	5,005E-06
FG-879	8,000E+02	1,171E-06	4,082E-08	3,735E-07	1,447E-07	2,741E-07	2,196E-08	1,578E-07	1,030E-07	1,516E-04	4,915E-06
FG-880	8,830E+02	1,194E-06	4,082E-08	5,438E-07	1,430E-07	2,762E-07	2,196E-08	1,794E-07	1,030E-07	1,529E-04	4,856E-06
FG-881	9,610E+02	1,245E-06	4,082E-08	4,817E-07	1,422E-07	2,872E-07	2,196E-08	1,356E-07	1,030E-07	1,664E-04	4,829E-06
FG-882	1,026E+03	1,324E-06	4,082E-08	2,852E-07	1,421E-07	3,052E-07	2,196E-08	6,268E-08	1,030E-07	1,924E-04	4,828E-06
FG-883	1,103E+03	1,479E-06	4,082E-08	3,172E-08	1,428E-07	3,404E-07	2,196E-08	-3,014E-08	1,030E-07	2,454E-04	4,850E-06

PAL12-16

Blanks		36Ar [V]	1σ	37Ar [V]	1σ	38Ar [V]	1σ	39Ar [V]	1σ	40Ar [V]	1σ
FG-1070	652 °C	1,193E-06	2,652E-08	1,361E-07	1,210E-08	2,533E-07	1,367E-08	1,107E-07	6,965E-08	2,287E-04	5,654E-06
FG-1071	707 °C	1,221E-06	2,615E-08	1,197E-07	1,210E-08	2,567E-07	1,367E-08	1,690E-07	6,965E-08	2,378E-04	5,576E-06
FG-1072	757 °C	1,249E-06	2,588E-08	1,166E-07	1,210E-08	2,567E-07	1,367E-08	1,753E-07	6,965E-08	2,499E-04	5,518E-06
FG-1073	811 °C	1,279E-06	2,567E-08	1,186E-07	1,210E-08	2,568E-07	1,367E-08	1,590E-07	6,965E-08	2,628E-04	5,474E-06
FG-1074	877 °C	1,319E-06	2,553E-08	1,207E-07	1,210E-08	2,609E-07	1,367E-08	1,359E-07	6,965E-08	2,776E-04	5,444E-06
FG-1075	944 °C	1,376E-06	2,551E-08	1,184E-07	1,210E-08	2,737E-07	1,367E-08	1,272E-07	6,965E-08	2,931E-04	5,438E-06
FG-1076	1017 °C	1,493E-06	2,564E-08	1,099E-07	1,210E-08	3,062E-07	1,367E-08	1,433E-07	6,965E-08	3,195E-04	5,466E-06

PAL12-17

Blanks		36Ar [V]	1σ	37Ar [V]	1σ	38Ar [V]	1σ	39Ar [V]	1σ	40Ar [V]	1σ
FG-1028	656 °C	1,292E-06	3,847E-08	1,387E-07	6,644E-07	2,948E-07	3,000E-08	1,004E-07	1,537E-07	2,132E-04	4,133E-06
FG-1029	724 °C	1,387E-06	3,847E-08	1,387E-07	6,535E-07	3,244E-07	3,000E-08	3,330E-07	1,537E-07	2,369E-04	4,065E-06
FG-1030	786 °C	1,453E-06	3,847E-08	1,354E-06	6,462E-07	3,460E-07	3,000E-08	7,274E-07	1,537E-07	2,661E-04	4,020E-06
FG-1031	846 °C	1,519E-06	3,847E-08	3,461E-06	6,417E-07	3,658E-07	3,000E-08	1,293E-06	1,537E-07	2,983E-04	3,992E-06
FG-1033	965 °C	1,705E-06	3,847E-08	1,009E-05	6,400E-07	4,103E-07	3,000E-08	2,932E-06	1,537E-07	3,647E-04	3,981E-06
FG-1034	1025 °C	1,829E-06	3,847E-08	1,440E-05	6,430E-07	4,371E-07	3,000E-08	3,974E-06	1,537E-07	3,961E-04	4,000E-06
FG-1035	1080 °C	1,952E-06	3,847E-08	1,870E-05	6,478E-07	4,637E-07	3,000E-08	5,007E-06	1,537E-07	4,224E-04	4,030E-06
